# Neuroleptic Malignant Syndrome Unmasked: A Case of Extrapyramidal Syndrome With Tardive Dystonia Leading to a Life-Threatening Crisis

**DOI:** 10.7759/cureus.97056

**Published:** 2025-11-17

**Authors:** Teresa Abegão, Mariana Antão, Cláudia Fitas, Nataliya Pylyp, Mariana Jordão

**Affiliations:** 1 Internal Medicine Department, Unidade Local de Saúde do Algarve - Hospital de Faro, Faro, PRT; 2 Psychiatry Department, Unidade Local de Saúde do Algarve - Hospital de Faro, Faro, PRT

**Keywords:** antipsychotic-induced complications, cotard's syndrome, dopamine antagonists, extrapyramidal syndrome, neuroleptic malignant syndrome, tardive dystonia

## Abstract

We present a 57-year-old male with a history of depression and Cotard’s syndrome, admitted to the psychiatric ward due to worsening psychotic symptoms. His antipsychotic regimen was intensified, including haloperidol, olanzapine, and promethazine. One month later, he developed a progressive akinetic-rigid syndrome, evolving into severe axial rigidity, autonomic instability, and hyperthermia. After 168 days of hospitalization, he was transferred to the emergency department in a stuporous state, with metabolic acidosis, rhabdomyolysis, acute kidney injury, and elevated creatine phosphokinase. A diagnosis of neuroleptic malignant syndrome (NMS) was established based on Levenson’s criteria. Immediate cessation of neuroleptics, aggressive supportive care, and treatment with bromocriptine, dantrolene, and benzodiazepines led to clinical improvement. Despite partial recovery, the patient remained with persistent dystonia and functional impairment.

This case emphasizes the importance of early recognition of NMS, especially in non-psychiatric settings, to prevent severe complications. Internists should be aware of extrapyramidal syndromes, particularly when evaluating patients on chronic antipsychotic therapy. A multidisciplinary approach, including neurologists, psychiatrists, and rehabilitation specialists, is crucial to optimizing patient outcomes and reducing long-term disability.

## Introduction

Neuroleptic malignant syndrome (NMS) is a rare entity, occasionally encountered by the psychiatrist, but not frequently found in Internal Medicine wards. Incidence is estimated to range from 0.02% to 3.23% among patients treated with antipsychotic medication. It is an idiosyncratic reaction to antipsychotic medication, especially the first-generation ones, such as haloperidol. First-generation antipsychotics (FGAs) are implicated in approximately 95% of all NMS cases [1-3]. High-potency FGA poses a greater risk of NMS, although all dopamine antagonists have been associated with the syndrome. It can occur with a single administration of the drug, or, although uncommon, after years of exposure to the same dose of the same antipsychotic [1,2].

Many factors have been identified as causative of increased risk of NMS, such as psychomotor agitation, parenteral administration of the antipsychotic, rapid dose increase, higher total daily drug dose, exhaustion, dehydration, and malnutrition. Previous episodes of NMS are a strong risk factor, increasing the likelihood of developing NMS after rechallenge with neuroleptics in 17-30% [1,2]. Although NMS can be diagnosed in virtually all ages of patients receiving antipsychotic treatment and in both sexes, young adult males predominate, especially those with intellectual disability or organic brain disease [1-3].

Clinical presentation is variable, but mental state changes (ranging from delirium to altered consciousness, from stupor to coma) are among the earliest signs. Generalized muscular rigidity, described as "lead pipe" rigidity, is a cardinal feature of NMS. Other neurological signs, including tremors, dysarthria, akinesia, sialorrhea, dysphagia, trismus, dystonia, hyperthermia, and, lastly, autonomic dysfunction, can present. Usually, rhabdomyolysis develops [1-3].

Differential diagnosis is of paramount importance, as NMS is a diagnosis of exclusion. Neuropsychiatric, systemic and drug-induced hypermetabolic diseases need to be ruled out - examples include central nervous system infections or lesions (stroke, cancer or abscesses), endocrinopathies (pheochromocytoma, thyrotoxicosis), autoimmune diseases (neuropsychiatric systemic lupus erythematosus or mixed connective tissue diseases), serotonin syndrome, heatstroke, drug intoxication (cocaine, amphetamines, phencyclidine), parkinsonian hyperthermia (following abrupt discontinuation of dopamine agonists used in Parkinson’s disease treatment), atropine poisoning from anticholinergics, and alcohol or sedative withdrawal [1-8].

In psychiatric patients, particularly those with schizophrenia or mood disorders, malignant catatonia may present with clinical manifestations indistinguishable from NMS. Both conditions share core features, including muscular rigidity, autonomic instability, altered consciousness, and hyperthermia, rendering differential diagnosis particularly challenging. This phenotypic overlap has significant therapeutic implications, as malignant catatonia may occur independently of antipsychotic exposure, whereas NMS represents an idiosyncratic adverse reaction to these agents [4-6].

No specific laboratory abnormality is diagnostic of NMS, but many support it. Patients exhibit leucocytosis (with or without left shift), metabolic acidosis, hypoxia, elevated serum creatine phosphokinase, myoglobinuria, and acute renal failure. Neuroimaging studies are generally unremarkable, as is cerebrospinal fluid analysis, but up to 54% of patients with NMS demonstrate nonspecific generalized slowing on electroencephalography [1-6]. Most of all, it is important to emphasize that NMS is a clinical diagnosis, and Levenson’s criteria provide a widely used framework for its identification - requiring all major criteria (fever, severe muscle rigidity and exposure to dopamine-blocking agent) and at least two minor criteria (diaphoresis, dysphagia, tremor, incontinence, altered level of consciousness, mutism, tachycardia, elevated or labile blood pressure, leukocytosis or elevated creatine phosphokinase) [4-8].

Mortality rates, initially reported as 76% in the first cases described in the 1960s, have significantly decreased to 10-20% in contemporary clinical practice. This improvement reflects advances in early recognition and management of the condition. Current fatalities primarily result from dysautonomic manifestations of the disease and systemic complications, including cardiovascular instability, respiratory failure, acute kidney injury, and thromboembolic events [3].

This case illustrates the diagnostic complexities encountered when NMS develops in a patient with severe psychiatric illness requiring prolonged antipsychotic exposure.

## Case presentation

We present the case of a 57-year-old Caucasian male with a previous diagnosis of depression and Cotard’s syndrome, who was completely independent in daily-life activities and lived independently. Previously treated with multiple psychotropic medications (risperidone 0.5mg once daily, olanzapine 10mg once daily, amisulpride 50mg once daily, clonazepam 2mg once daily, clomipramine 75mg once at breakfast and 150mg at dinner, olanzapine 10mg once daily if anxiety or insomnia), the patient was admitted to the psychiatry ward due to worsening of the psychotic symptoms, with depressed mood and nihilist delirium.

During his stay, medication was adjusted to venlafaxine 150mg at breakfast, clomipramine 75mg at breakfast and 150mg at dinner, quetiapine 300mg at night, haloperidol 5mg twice daily, olanzapine 10mg once daily if anxiety or insomnia, promethazine 50mg, and diazepam 10mg intramuscular up to every eight hours as needed for agitation.

One month after admission, the patient developed symptoms compatible with an akinetic-rigid syndrome - starting with difficulties in balance and autonomous mobility, progressing to inability to stand unassisted or walk, which confined him to a wheelchair, with consequent muscle stiffness, flexion of both hands and fingers, unable to feed autonomously - which was initially attributed to his psychiatric decompensation. Due to maintenance of the psychotic syndromes despite dose escalation, the patient started electroconvulsive therapy (ECT), completing a total of eight sessions. In the days preceding evaluation by the internal medicine team, the patient exhibited increasing agitation, leading to an increase in the dosage of medications (haloperidol, olanzapine, and promethazine).

After 168 days of admission, he was transferred to the general emergency department, presenting with acute altered mental status, dyspnoea, and fever. At admission, the patient was stuporous (Glasgow Coma Scale of 7 points), tachycardic (heart rate of 95-100 beats per minute), with a peripheral oxygen saturation of 98% with a venturi oxygen mask with FiO_2_ 31%, temperature of 39ºC, flexion of both hands and feet, and axial rigidity. The admission blood tests showed leukocyte count at the upper limit of normal (10x10^9^/L), and a mildly elevated C-reactive protein (CRP 14mg/L), with altered renal function (creatinine 1.5mg/dL). The chest radiography showed no evidence of pneumonia. The assumed diagnosis was tracheobronchitis, and the patient was started on empirical antibiotics (with ceftriaxone 1g every 12 hours) and kept on all the medication prescribed by the psychiatrist. Overnight, the patient had agitation periods, with the need for administration of additional doses of haloperidol 5mg IM twice, chlorpromazine 300mg, and olanzapine 10mg.

The next day, upon transfer to our internal medicine service, we found the patient with a fever of 39-40ºC that was poorly responsive to antipyretics, with progressively shorter afebrile intervals (four hours). The patient had a labile blood pressure, with systolic blood pressure values ranging from 90 to 140mmHg in both arms. His neurological status showed no improvement, and he remained stuporous, mute, with axial rigidity and flexion of both hands and feet (as shown in Videos 1-2), with brisk and symmetrical deep tendon reflexes and bilateral flexor plantar responses, incontinent and diaphoretic.

**Video 1:** Lower Limb Examination in Neuroleptic Malignant Syndrome (NMS) This video demonstrates a clinical examination of the lower extremities in a patient with NMS upon admission to the emergency department. At rest, the patient exhibits plantar flexion of the ankle. Note the severe axial rigidity that prevents passive flexion of the legs. This lead-pipe rigidity is characteristic of NMS and is non-velocity dependent, distinguishing it from spasticity. The rigidity persists throughout the range of motion attempt and is a cardinal feature of this potentially life-threatening condition. The patient provided written consent to allow the publication of his images or videos.

**Video 2:** Upper Limb Examination in Neuroleptic Malignant Syndrome (NMS) This video demonstrates the examination of the upper extremities in the same patient with NMS upon admission to the emergency department. At rest, the patient demonstrates wrist flexion. Similar to the lower limbs, there is prominent axial rigidity that impedes passive movement of the arms. The examiner is unable to flex the patient's arms due to this severe muscular rigidity, which is consistent throughout the attempted range of motion. This generalized rigidity, affecting both upper and lower extremities, is typical in advanced cases of NMS. The patient provided written consent to allow the publication of his images or videos.

Pulmonary auscultation was clear, and the peripheral oxygen saturation was 100% with a venturi oxygen mask with FiO_2_ 31%. Arterial blood gas analysis showed a metabolic acidosis with hyperlacticaemia (pH 7.27, partial pressure of carbon dioxide of 22mmHg, bicarbonate 9.9mmol/L, lactates 7.9mmol/L), severe hypocalcaemia (Ca 0.99mmol/L), and no hypoxemia (partial pressure of oxygen of 112mmHg). The patient was oliguric (diuresis of 100mL in eight hours, equivalent to 0.1mL/Kg/hour).

Laboratory investigations revealed severe rhabdomyolysis, acute kidney injury, hepatocellular injury, and systemic inflammation; thyroid function tests and autoimmune/infectious serologies were unremarkable (Table 1).

**Table 1:** Laboratory Findings on Hospital Admission

Parameter	Value	Reference Range
Leucocytes	18.2x10^9^/L	4-10x10^9^/L
Neutrophils	16.4x10^9^/L	2-7x10^9^/L
C-reactive protein	24mg/dL	<5mg/dL
Creatine phosphokinase	21977UI/L	30-200UI/L
Creatinine	3.5mg/dL	0.7-1.3mg/dL
Blood urea nitrogen	83g/dL	8.4-25.7g/dL
Calcium	6.6mg/dL	8.4-10.2mg/dL
Phosphorus	8.6mg/dL	2.3-4.7mg/dL
Aspartate aminotransferase	5692UI/L	5-34UI/L
Alanine aminotransferase	9876UI/L	<55UI/L
International normalized ratio	2.5	-
Thyroid-stimulating hormone	2.76μIU/mL	0.35-4.94μIU/mL
Free thyroxine	1.06ng/dL	0.93-1.70ng/dL
Antinuclear antibodies	Negative	-
Herpes simplex virus IgM and IgG	Negative	-
Human immunodeficiency virus IgM and IgG	Negative	-
Treponema pallidum IgM and IgG	Negative	-
Cytomegalovirus IgM and IgG	Negative	-

Cerebrospinal fluid analysis obtained through lumbar puncture yielded negative results (Table 2).

**Table 2:** Cerebrospinal Fluid Analysis PCR: polymerase chain reaction; HSV: herpes simplex virus; HHV-6: human herpesvirus 6; VZV: varicella-zoster virus; CMV: cytomegalovirus

Parameter	Value	Reference Range
Leucocytes	2cells/mm^3^	<6cells/mm^3^
Glucose	62mg/dL	40-70mg/dL
Proteins	38mg/dL	15-60mg/dL
Lactate	1.8mmol/L	0.6-2.2mmol/L
Gram stain and bacterial culture	Negative	-
PCR testing for common meningitis and encephalitis pathogens (HSV-1/2, VZV, enterovirus, HHV-6, parechovirus, CMV, *Neisseria meningitidis, * *Streptococcus pneumoniae, * *Haemophilus influenzae, Escherichia coli* K1, *S. agalactiae, Listeria monocytogenes, *and* Cryptococcus neoformans*)	Negative	-

A head CT scan was requested, and no abnormalities were detected, confirmed by a later head MRI. Although the patient was already on antibiotics, we decided to request two sets of blood cultures, which remained sterile. Electroencephalogram excluded status epilepticus and other pathologic activity.

Based on the patient’s history and physical examination, the most likely diagnosis was an extrapyramidal syndrome with tardive dystonia secondary to neuroleptics, and later NMS secondary to the increased dosage of antipsychotics, fulfilling all major criteria and multiple minor criteria of Levenson's diagnostic criteria.

The patient was immediately started on intensive fluid replacement with sodium bicarbonate and sodium chloride 0.9%, correction of electrolyte imbalances (especially hypocalcaemia), and all psychiatric medication was discontinued. Bromocriptine 2.5mg every six hours and dantrolene 160mg followed by 80mg every six hours and diazepam 10mg every eight hours were started. The patient was admitted to the Intensive Care Unit (ICU) for stabilization and surveillance. His symptoms started to improve within the following days, with an improvement in the Glasgow Coma Scale score to 13-14, resolution of the rhabdomyolysis, and normalization of the renal function and liver enzymes.

After four days in the ICU, the patient was transferred to an Internal Medicine ward, where he remained for 18 days. Rehabilitation was started, aiming to decrease the level of spasticity and recovery of functional autonomy. After that period of time, he was transferred back to the Psychiatric ward, where ECT was resumed. During his two-month stay in the ward, he showed minimal functional improvement, being able only to sit in a chair with help from a third party, unable to walk, dysarthric, with persistent cervical rigidity, bilateral dystonic hand posturing with wrist flexion, and bilateral dystonic foot posturing with severe, minimally reducible equinovarus deformity.

Following discharge to a rehabilitation unit, the patient began regular psychiatric follow-up, which continues to the present day. His psychiatric condition is stable, and physically, he regained function in his right hand, still requiring assistance with activities of daily living and a wheelchair for mobility.

## Discussion

NMS is an idiosyncratic reaction that primarily affects patients receiving FGAs, though all dopamine antagonists can be implicated. It is associated with dopamine blockade, particularly in cases with high-potency FGAs, rapid dose escalation, dehydration, or prior NMS episodes. The pathophysiology involves dopamine D2 receptor antagonism leading to central nervous system dysfunction, autonomic instability, hyperthermia, and severe muscle rigidity, which may progress to rhabdomyolysis and multi-organ failure if not promptly treated. The disorder requires astute clinical recognition, as no single test can confirm NMS definitively [5].

Our case describes a 57-year-old male with a psychiatric history who developed progressive motor symptoms and, later, a complete NMS presentation, fulfilling all of Levenson’s criteria. Early extrapyramidal symptoms were not recognized due to attribution to his underlying Cotard's syndrome, resulting in continued antipsychotic exposure and progressive clinical deterioration until critical intervention was required.

Diagnosing NMS in psychiatric patients with psychotic symptoms poses a significant challenge due to difficulty distinguishing symptoms arising from the underlying psychiatric disorder from medication-related adverse effects. This diagnostic complexity is further heightened in cases of Cotard’s syndrome, a rare neuropsychiatric syndrome characterized by nihilistic delusions in which patients believe they are dead, do not exist, or lack internal organs. The profound psychomotor retardation, mutism, and catatonic features that may accompany Cotard's syndrome may closely mimic NMS symptoms, potentially delaying recognition and timely intervention. Moreover, patients with treatment-resistant Cotard's syndrome frequently require antipsychotic polypharmacy, which increases their risk of developing NMS. This phenotypic overlap underscores the importance of meticulous clinical assessment, comprehensive medication review, and heightened vigilance for early signs of NMS to distinguish NMS from underlying psychiatric manifestations and prevent potentially life-threatening complications.

This case underscores the importance of a multidisciplinary approach involving psychiatry, internal medicine, and intensive care specialists to ensure prompt identification and management. The delayed discontinuation of neuroleptics in this patient likely contributed to the progression of his clinical deterioration, highlighting the critical importance of recognizing early extrapyramidal symptoms as potential harbingers of NMS.

Furthermore, this case critically highlights the role of ECT as a potential iatrogenic diagnostic obstacle. Although ECT is a well-established treatment for malignant catatonia, which shares a phenotypic overlap with NMS, its utility as a first-line therapy for NMS remains ambiguous. In our patient, ECT was initiated (eight sessions) despite the progression of early motor symptoms (akinetic-rigid syndrome) and prior to a differentiated diagnosis. This intervention may have masked the worsening of NMS, aggravated diagnostic uncertainty by focusing treatment on catatonia, and consequently significantly delayed the critical discontinuation of neuroleptics and the implementation of the most appropriate intervention protocol for NMS.

## Conclusions

This case highlights the critical importance of early recognition of NMS, especially in psychiatric patients receiving multiple antipsychotics. The diagnostic challenge is compounded in conditions such as Cotard's syndrome, where underlying psychiatric symptoms may obscure early extrapyramidal manifestations. Clinicians must maintain heightened clinical suspicion for NMS in patients developing new-onset rigidity, autonomic instability, or altered mental status whilst on dopamine antagonists. Prompt discontinuation of neuroleptics combined with intensive supportive care is essential in minimizing morbidity and mortality. A collaborative multidisciplinary approach involving psychiatry, internal medicine, and critical care specialists is crucial for optimal outcomes, with early recognition and prompt intervention being paramount to prevent severe, potentially irreversible sequelae of this life-threatening condition.
